# Efficacy of anlotinib and chemotherapy combination as neoadjuvant therapy in the treatment of pulmonary artery intimal sarcoma: a case report

**DOI:** 10.3389/fonc.2025.1507281

**Published:** 2025-02-26

**Authors:** Gai Liang, Qu Zhang, Yan Luo, Yuanhua Zhao, Bo Luo

**Affiliations:** Department of Radiation Oncology, Hubei Cancer Hospital, TongJi Medical College, Huazhong University of Science and Technology, Wuhan, China

**Keywords:** pulmonary artery intimal sarcoma, anlotinib, neoadjuvant treatment, chemotherapy, case report

## Abstract

Pulmonary arterial intimal sarcoma (PAIS) is a rare malignant mesenchymal tumor often associated with an unfavorable prognosis and lacks a standardized treatment approach to date. This report presents a notable case of PAIS treated with neoadjuvant therapy involving anlotinib concomitantly administered with chemotherapy of ifosfamide and pirarubicin, which resulted in a favorable outcome. A 38-year-old man was admitted to our hospital with chest tightness, cough, and dyspnea, all of which had persisted for more than a week. Initial evaluation via chest computed tomography (CT) revealed a sizable posterior mediastinal tumor measuring 11.9 × 7.6 cm. A CT-guided biopsy was performed, and pathological findings confirmed the diagnosis of PAIS. Efficacy evaluation showed slow progress after one cycle of chemotherapy with ifosfamide and pirarubicin. To enhance treatment outcomes, we incorporated anlotinib as a neoadjuvant therapy alongside ifosfamide and pirarubicin. Subsequent CT imaging demonstrated a significant reduction in tumor size, and the patient experienced notable alleviation of symptoms. The patient then underwent surgery, radiation, and subsequently, maintenance treatment with anlotinib for one year. No severe drug-related side effects were observed. The patient achieved progression-free survival of 25 months following administration of anlotinib. Thus, the combination of anlotinib with ifosfamide and pirarubicin demonstrated significant efficacy and safety. This approach holds promise as an effective therapeutic strategy for managing unresectable, locally advanced, or advanced PAIS. However, further clinical studies are necessary to validate these findings.

## Introduction

Pulmonary arterial intimal sarcoma (PAIS) is a rare malignant mesenchymal tumor that occurs in large blood vessels of the pulmonary circulation (1). PAIS can originate from the left and right pulmonary arteries, as well as the intimal layer of the pulmonary arteries, forming a tumor that either grows in a nodular cavity or spreads along the intimal surface (2). The prognosis of PAIS is poor, and currently, there is no established standard treatment strategy (3). Surgical resection is presently the primary choice of treatment for PAIS, and the role of postoperative radiotherapy and chemotherapy remains controversial (4). However, there have been no reports on the treatment of PAIS using a neoadjuvant regimen with anlotinib. In this study, we present a noteworthy case of a patient with PAIS who underwent neoadjuvant treatment using the anlotinib and ifosfamide and pirarubicin regimen, resulting in a successful outcome. The combination therapy for neoadjuvant treatment may lead to a better PAIS prognosis.

## Case/case series presentation

**Figure 1:**
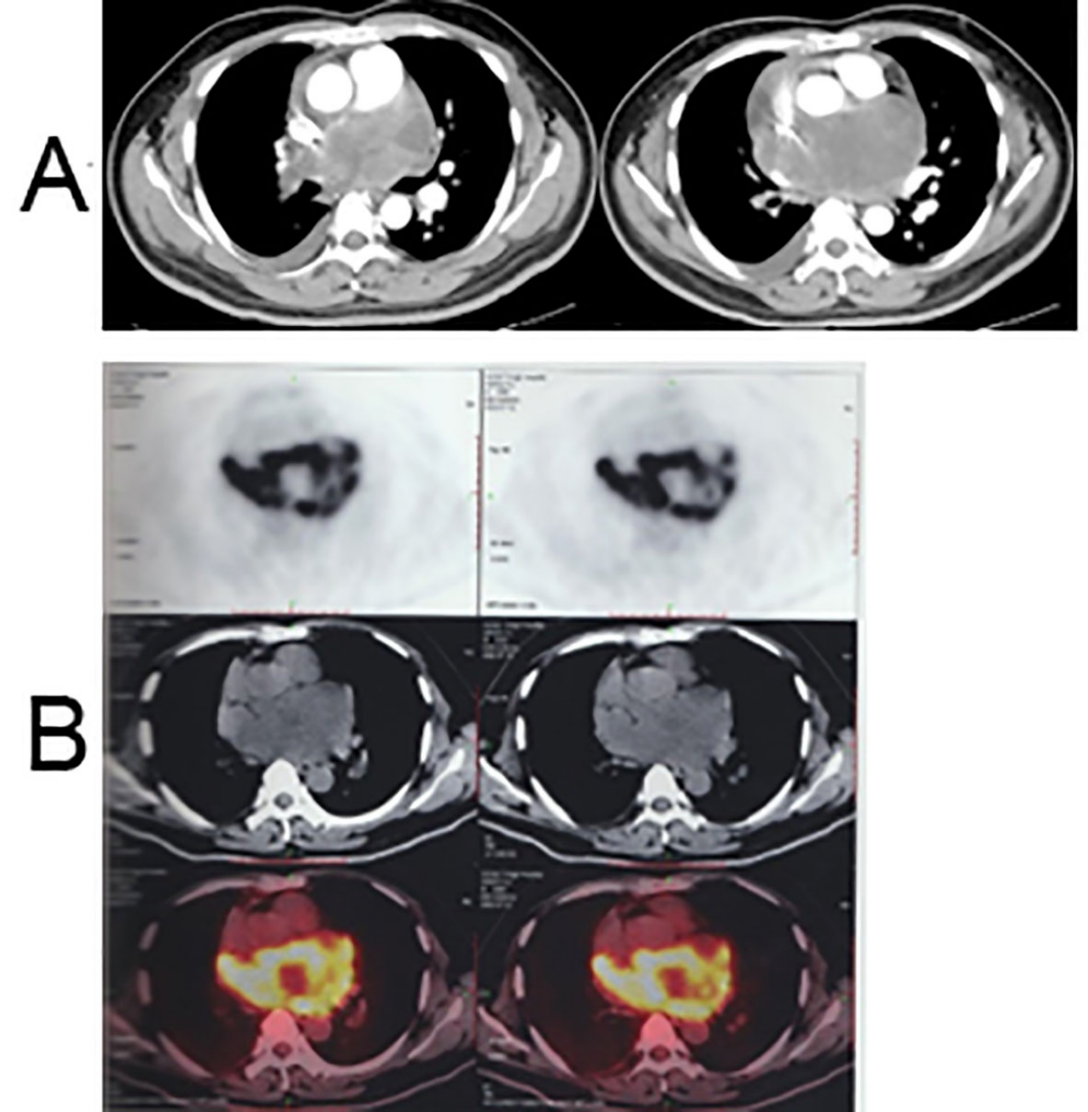
Chest CT and PET-CT scan before treatment. **(A)** Enhanced chest CT scan images: a posterior mediastinal tumor measuring 11.9 × 7.6 cm with involvement of the right pulmonary arteries, accompanied by pericardial effusion and minor pleural effusion on both sides; **(B)** PET-CT scan image: a large soft tissue mass in the posterior mediastinum with increased metabolism (SUV_max_: 26.3), involving the right pulmonary artery trunk and associated arteries.

In June 2022, a 38-year-old man was admitted to our hospital with chest tightness, cough, and dyspnea, all of which had persisted for more than a week. Physical examination of the patient revealed a body temperature of 36.6°C, blood pressure of 95/78 mmHg, tachycardia characterized by a heart rate of 120 bpm, and tachypnea with a respiratory rate of 24 bpm. The oxygen saturation (SpO_2_) in room air was recorded at only 98%. Liver function tests, renal function tests, and complete blood count showed normal results. He had no medical, family, psychosocial, or genetic history. Upon assessment, the Eastern Cooperative Oncology Group (ECOG) performance status score was determined to be 2. On July 4, 2022, an initial chest computed tomography (CT) evaluation revealed a sizable posterior mediastinal tumor measuring 11.9 × 7.6 cm. This tumor not only involved the right pulmonary arteries but also led to pericardial effusion, accompanied by a minor pleural effusion on both sides (**Figure 1A**). Further positron emission tomography-computed tomography (PET-CT) investigations revealed a large soft-tissue mass with increased metabolic activity (SUV_max_: 26.3) within the posterior mediastinum. Notably, this mass involved the right pulmonary artery trunk and its associated arteries, prompting considerations of a potential malignant tumor (**Figure 1B**).

**Figure 2:**
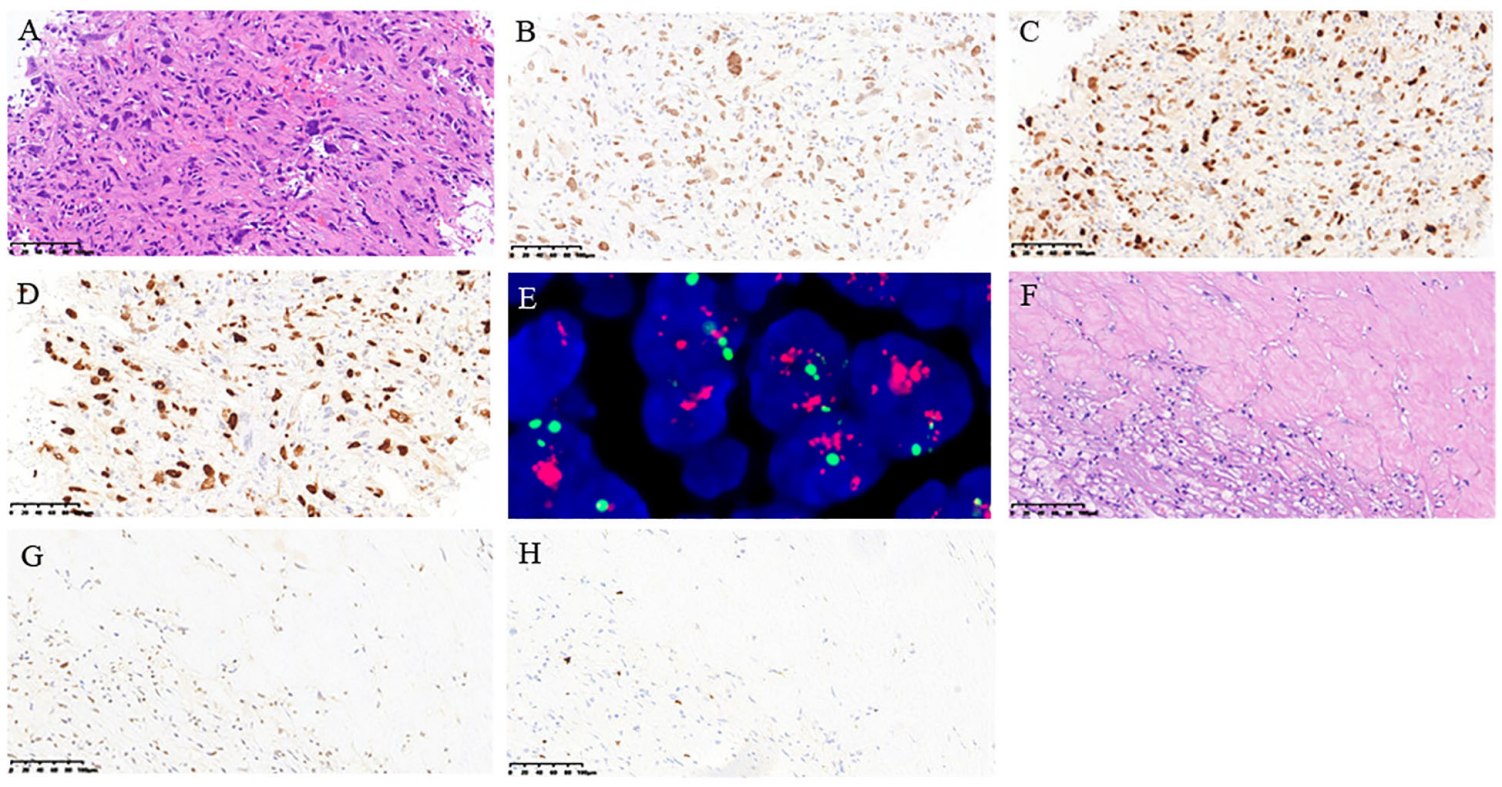
Pathological findings of the histology of posterior mediastinal biopsy tissue. **(A)** H&E staining; **(B)** immunohistochemical staining of SATB2; **(C)** immunohistochemical staining of MDM2; **(D)** immunohistochemical staining of Ki67 index; **(E)** fluorescence *in situ* hybridization detection of MDM2; **(F)** H&E staining; **(G)** immunohistochemical staining of MDM2; **(H)** immunohistochemical staining of Ki67. (**A-E**: From the biopsy sample before treatment, **F-H**: from the surgical sample after treatment).

After discussions with specialists in thoracic surgery, medical oncology, radiation oncology, and radiology during a multidisciplinary treatment (MDT) consultation, we concluded that complete tumor resection was not feasible in this case. Therefore, a CT-guided biopsy was performed on July 18, 2022. Pathological findings revealed spindle or epithelioid shapes in the tumor cells, characterized by abundant cytoplasm and large nuclei with rough chromatin. Positive immunohistochemical staining for SATB2 and MDM2 was observed, along with a high proliferative index (Ki67). Amplification of MDM2 was confirmed through fluorescence *in situ* hybridization detection (**Figures 2A–E**). Combining the medical history, clinical imaging, immunohistochemistry, and molecular test analyses, we established a diagnosis of PAIS with the clinical stage T3N0M0 (IIIB) according to the eighth edition of the AJCC cancer staging.

**Figure 3:**
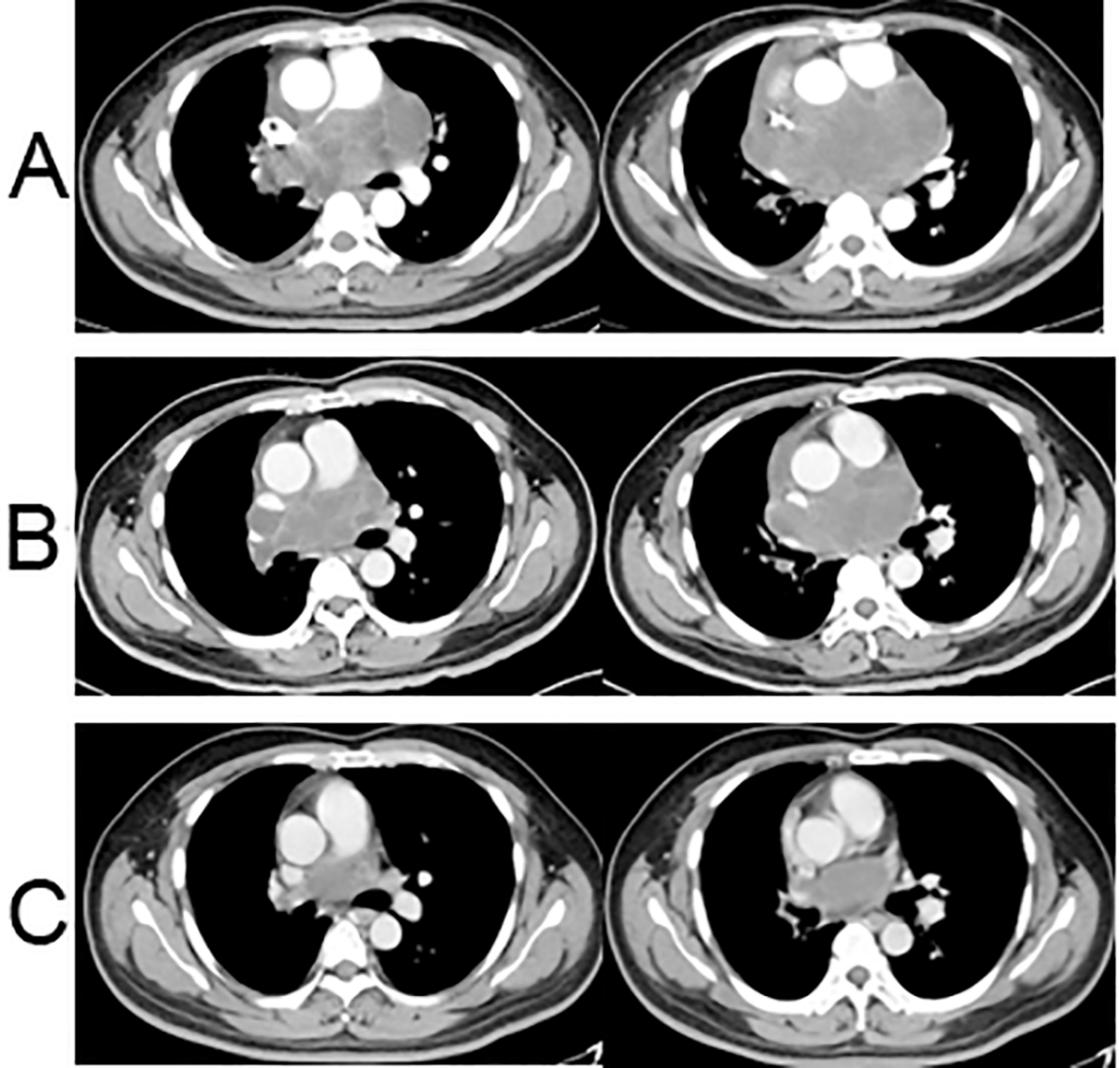
Chest CT scans after neoadjuvant treatment. **(A)** Chest CT enhanced scan after one cycle of chemotherapy: the tumor had increased in size, measuring 14.0 × 7.5 cm; **(B)** Chest CT enhanced scan after two cycles of chemotherapy + anlotinib: the tumor had significantly shrunk with a size of approximately 10.1 × 6.3 cm; **(C)** Enhanced chest CT scan after five cycles of chemotherapy + anlotinib: the tumor had significantly shrunk at the end of the fifth cycle of chemotherapy + anlotinib, measuring 6.8 × 3.7 cm.

According to pathological findings, the patient underwent one cycle of chemotherapy using the ifosfamide and pirarubicin (ifosfamide, 2 g/m^2^, days 1-5; pirarubicin, 40 mg/m^2^, day 1) on July 22, 2022. Three weeks later, despite the treatment, the patient’s chest pain and cough persisted. A follow-up CT scan indicated that the tumor had not diminished significantly and measured 14.0 × 7.5 cm. The efficacy evaluation progressed slowly but did not reach PD (**Figure 3A**). As the effect of chemotherapy alone proved unsatisfactory, and based on insights from prior clinical studies suggesting improved efficacy with antiangiogenic agents, the decision was made to incorporate anlotinib into the treatment plan. Anlotinib, which is known to improve the survival rates of patients with advanced soft tissue sarcomas (5), was prescribed at a dosage of 12 mg once daily for 2 weeks, followed by a 1-week break, in combination with ifosfamide and pirarubicin. After two cycles of administration, a subsequent CT scan revealed a significant reduction in tumor size, measuring approximately 10.1 × 6.3 cm (**Figure 3B**). Encouraged by the positive response, the patient continued treatment with a combination of anlotinib and chemotherapy with ifosfamide and pirarubicin for five cycles. The final CT scan at the end of the last cycle showed a substantial reduction in tumor size, measuring 6.8 × 3.7 cm. Clinically, the evaluation was a partial response (**Figure 3C**), and the patient experienced gradual relief from chest tightness, dyspnea, and cough. Throughout the treatment process, the patient experienced some adverse effects, including grade 1 nausea and vomiting, grade 1 hypertension, grade 3 leukopenia, and grade 1 rash.

Subsequently, the tumor stabilized at a size of 6.8 × 3.7 cm. Following a second MDT discussion, the patient was advised to undergo surgery for palliative tumor reduction. He consented to this treatment approach and underwent the resection of the remaining tumor on January 11, 2023.

Postoperative pathology revealed the following findings: a treatment-induced tumor reaction marked by the degeneration and necrosis of tumor cells, proliferation of stromal fibrous tissue, and infiltration of foam cells and mixed inflammatory cells. The remaining limited tumor cells still expressed MDM2 but exhibited a low Ki67 (**Figures 2F–H**).

**Figure 4:**
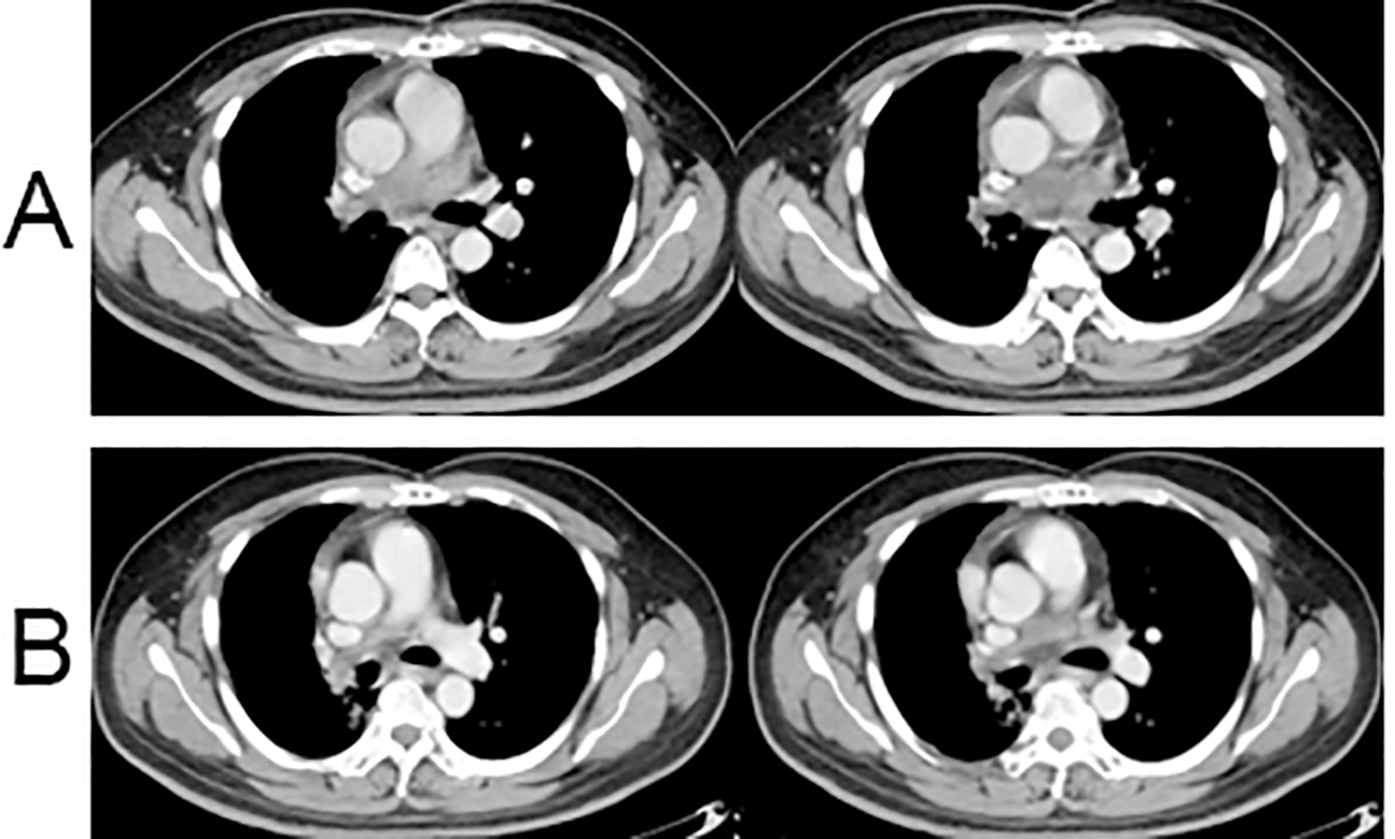
Postoperative chest CT results. **(A)** Enhanced chest CT scan at 1 month after surgery; **(B)** Enhanced chest CT scan at 6 months after surgery.

One month post-surgery, the CT scan indicated normal postoperative changes, and the tumor size had decreased from 6.8 × 3.7 cm to 3.5 × 2.7 cm (**Figure 4A**). The patient then underwent intensity-modulated radiotherapy at a total dose of 66 Gy in 33 fractions. The planning target volume included the residual tumor and tumor bed. Throughout the radiotherapy sessions, the patient concurrently received maintenance treatment with anlotinib. After the completion of radiotherapy, the patient continued consolidation therapy with anlotinib for one year.

**Figure 5:**
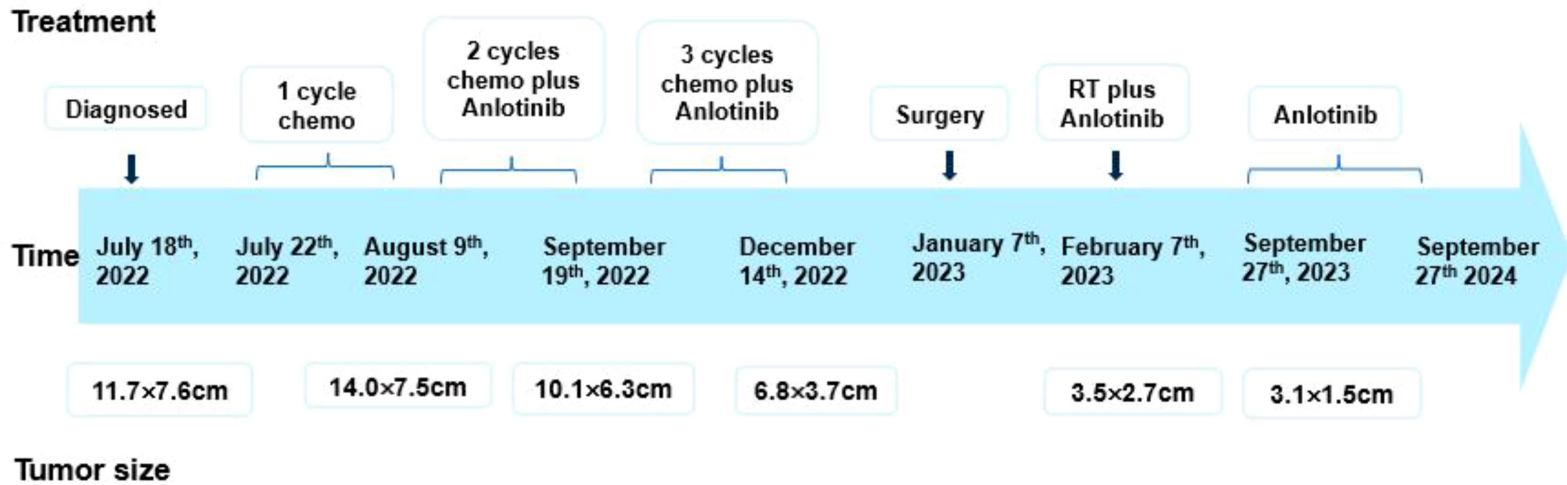
Timeline of tumor size and treatment of the patient. Chemo, ifosfamide and pirarubicin; RT, radiation therapy.

Clinical evaluation at the 8-month post-surgery mark revealed stable disease with a tumor size of 3.1 × 1.5 cm (**Figure 4B**). No severe drug-related side effects were observed. The timeline of changes in tumor size and treatments administered are shown in **Figure 5**. The patient achieved a progression-free survival (PFS) of 25 months as of September 27, 2024, and regular follow-up is being conducted.

## Discussion

PAIS is an extremely rare malignant mesenchymal tumor. As of 2023, approximately 400 cases of PAIS have been reported in the literature (3), most of which have been published as case reports. Despite this, the pathogenesis of the disease remains unclear. Common symptoms of PAIS include dyspnea, cough, chest tightness, and hemoptysis, often attributed to chronic right heart failure or associated malignant tumors (6). In the present case, the patient presented with difficulty in breathing, cough, and chest tightness associated with a malignant tumor.

The pathological characteristics of PAIS include predominantly spindle-shaped cells under light microscopy, along with scattered multinucleated giant cells and epithelioid cells. Immunohistochemistry lacks specificity, with most cases showing diffuse positivity for vimentin, along with partial expression of SMA and CD34 (7). Literature reports indicate frequent detections of PAIS based on gene amplifications, including MDM2 (65%), cyclin-dependent kinase 4, platelet-derived growth factor receptor α (81%), and EGFR (76%) (8). In the present case, MDM2 gene amplification was positive, aligning with findings in previous studies (3, 7).

Currently, no standardized treatment strategy exists for PAIS, particularly for cases that are unresectable or locally recurrent. Surgery remains the cornerstone of treatment for this condition, which may include pulmonary endarterectomy, lobectomy, or pneumonectomy. The role of adjuvant therapy is not well-defined; however, there are case reports indicating improved outcomes with the use of chemotherapy and/or radiation therapy (RT) (9). A review from the MD Anderson Cancer Center revealed a median survival of 25 months for patients receiving multimodal therapy, compared to just 8 months for those undergoing single-modality treatment (10). Most adjuvant chemotherapy regimens included anthracyclines, such as doxorubicin, as well as ifosfamide. Additionally, a limited number of studies have reported the use of gemcitabine and paclitaxel, although their efficacy appears to be suboptimal.

Neoadjuvant therapy involves the use of systemic treatments before surgery. Initially employed to treat inoperable locally advanced breast cancer (11), neoadjuvant therapy has proven effective in increasing the likelihood of breast-conserving surgery and thus established itself as a viable option for patients with operable disease (12, 13). Studies have shown that patients who achieved a pathologic complete response after neoadjuvant treatment had a significantly better prognosis than those with residual disease (14–16). For unresectable or recurrent focal sarcomas, common neoadjuvant therapies involve chemotherapy with doxorubicin and cyclophosphamide, either alone or in combination with radiotherapy (17, 18). However, there have been no studies on neoadjuvant chemotherapy with antiangiogenic drugs for PAIS.

Anlotinib is a multi-target tyrosine kinase inhibitor with dual effects: inhibiting angiogenesis and directly impeding tumor growth. The functional targets of anlotinib encompass vascular endothelial growth factor receptors 1/2/3, platelet-derived growth factor receptor, fibroblast growth factor receptor, and c-Kit (19). Anlotinib’s mechanism of action includes inhibition of tumor vascular survival, normalization of tumor tissue blood vessels, improvement in tumor hypoxia, increase in chemotherapy distribution in tumor tissue, and further enhancement of anti-tumor effects. Therefore, the combination of anlotinib and chemotherapy can have a synergistic effect (20). Research has shown that in second-line advanced soft tissue sarcoma, anlotinib has an efficacy rate of 12.7%, with a PFS rate of 86.4% at 12 weeks. Results from the Phase IIB Alter0203 study reveal that, compared to a placebo, anlotinib significantly prolongs the time without disease progression and reduces the risk of progression in patients (6.27 months vs 1.47 months, HR=0.33) (5, 21). Notably, pathological types in this study mainly included leiomyosarcoma, synovial sarcoma, acinar soft tissue sarcoma, clear cell sarcoma, epithelioid sarcoma, undifferentiated pleomorphic sarcoma, liposarcoma, and fibrosarcoma and did not include PAIS. In the present case, clinical evaluation progressed slowly after one cycle of chemotherapy with ifosfamide and pirarubicin. The patient was dissatisfied with the efficacy of chemotherapy alone and continued to experience symptoms of cough and chest tightness. Following extensive communication with the patient, we decided to administer anlotinib in combination with ifosfamide and pirarubicin. After two treatment cycles, we observed a significant improvement in the patient’s symptoms of cough and chest tightness, reflected in the clinical evaluation as a partial response. Consequently, the patient continued this combined regimen for three cycles. The tumor size was effectively reduced with the combination treatment, leading to an improvement in the patient’s overall condition.

After the final chemotherapy session, a follow-up CT scan revealed a residual tumor in the right pulmonary artery, and the patient was intolerant to further chemotherapy. Following an MDT discussion, we recommended local surgery and radiotherapy. Postoperative pathology closely resembled complete remission. To minimize local recurrence and distant metastases, radiation therapy was administered for residual lesions after surgery concurrently with anlotinib therapy. As demonstrated in a clinical trial investigating the safety and efficacy of anlotinib, the most common adverse drug reactions (ADRs) associated with anlotinib include hypertension, hand-foot syndrome, and hyperlipidemia, among others (5). The patient exhibited good tolerance with no serious adverse effects.

The prognosis for patients with PAIS is generally poor, with untreated patients having an overall survival of approximately 1.5 to 3 months (4). The median survival after complete surgical resection can extend to 36.5 ± 20.2 months, whereas after incomplete surgical resection, it can reach 11.3 months (10). Funatsu (22) reported a partial response of PAIS to pazopanib as second-line treatment in a case with PFS of 4 months, and the patient required cessation of pazopanib because of severe hand-foot syndrome. Kollár’s study showed that pazopanib had promising activity as a second-line treatment in angiosarcoma with PFS of 3 months and OS of 9.9 months (23). Therapeutic interventions aimed at MDM2-amplified sarcoma are presently being evaluated in clinical trials. Takafumi noted that milademetan, an MDM2 inhibitor, showed effectiveness in patients with MDM2-amplified intimal sarcoma. This suggests it could be a viable treatment option for intimal sarcoma, as indicated by a Phase Ib/II study that reported PFS of 4.7 months, which merits further investigation (24).

In this case, the patient achieved a PFS of 25 months after treatment with anlotinib and maintained a good condition during follow-up. Such kind of multimodal therapy for PAIS may be a viable strategy. However, these data from the case report are still very limited, and further research is needed to elucidate the value of neoadjuvant chemotherapy with anlotinib for PAIS to improve the prognosis of patients.

## Conclusion

In summary, neoadjuvant treatment using the combination of anlotinib and chemotherapy with ifosfamide and pirarubicin successfully reduced the tumor size in a patient with PAIS, demonstrating favorable efficacy and safety. The combination of anlotinib and chemotherapy as a neoadjuvant therapy could be an effective strategy for unresectable locally advanced or advanced PAIS. However, further clinical studies are necessary to validate these findings.

## Data Availability

The raw data supporting the conclusions of this article will be made available by the authors, without undue reservation.
